# The Big Bang: A Virtual Subarachnoid Hemorrhage Simulation for Preclinical Medical Students

**DOI:** 10.7759/cureus.14919

**Published:** 2021-05-09

**Authors:** Katie M Harris, Gillian Sheppard

**Affiliations:** 1 Faculty of Medicine, Memorial University of Newfoundland, St. John's, CAN; 2 Emergency Medicine, Memorial University of Newfoundland, St. John's, CAN

**Keywords:** virtual simulation, simulation based medical education, subarachnoid hemorrhage, preclinical medical students

## Abstract

Simulation-based learning is important for rare, high mortality cases, which are unlikely to be witnessed during clinical rotations but are likely to be encountered during future practice such as a subarachnoid hemorrhage. Neurology case simulations, especially those targeted at preclinical learners, are underrepresented in simulation pedagogy, and preclinical learners are underrepresented in a meta-analysis of the efficacy of simulation-based medical education. We designed a virtual simulation of subarachnoid hemorrhage for preclinical medical students, which can be implemented during restricted access to clinical learning. The simulation is 15 minutes long and requires only one standardized patient and one evaluator, which makes this simulation accessible to institutions with limited simulation resources. We adapted the validated questions from the “Simulation Evaluation Tool - Modified” for our post-simulation survey, which will detect the students’ level of confidence and their perceived learning post-simulation. The analysis of student experiences using this validated tool will contribute to the literature base surrounding the efficacy of virtual simulation as a training tool for preclinical learners.

## Introduction

Map

A subarachnoid hemorrhage (SAH) occurs when blood moves into the subarachnoid space due to a compromise in a cerebral artery wall. As the blood diffuses into the subarachnoid space, symptoms may appear to resolve. This, in combination with the broad differential diagnosis for SAH, which includes ischemic stroke, meningitis, primary headaches syndromes, venous thrombosis, and drug toxicity among others, can lead to SAH being missed entirely or misdiagnosed [1]. A subarachnoid hemorrhage is misdiagnosed in 20%-50% of patients at first presentation, and failure to diagnose is associated with rebleeding in 15% of patients on the first day and 40% in the next four weeks [2]. If the diagnosis is overlooked, it results in up to half of the patients dying within three weeks of a SAH, and one-third living with severe cognitive impairment [2]. Therefore, a subarachnoid hemorrhage is considered one of the most difficult cases for emergency medicine practitioners [3].

A subarachnoid hemorrhage is an ideal case for simulation-based teaching due to the ambiguity of the presenting symptoms and the high associated risk with an inaccurate diagnosis [4]. We have developed a virtual subarachnoid hemorrhage simulation for training preclinical students to recognize the ‘red flags’ of a headache before entering clerkship, so that they are better prepared to recognize neurosurgical emergencies during their rotation and future practice and react appropriately. This simulation also addresses a critical gap in exposure, as during clerkship, medical students may not be exposed to conditions such as SAH, which are rare enough to be missed during rotation but common enough to appear in future practice [5].

Preclinical medical students consistently report that they enjoy simulation-based learning more than other teaching techniques [5-8], and there is significant evidence that simulation-based learning improves skill, confidence, internal motivation, and long-term recall [4]. By simulating SAH early in medical training, future doctors will be better prepared to recognize the signs of this challenging diagnosis.

Gap

Neurology simulations are severely underrepresented in Canadian medical school curriculums, as the traditional teaching of neurological emergencies is often through non-authentic learning environments such as classroom lectures [4,8]. At the time of writing, no subarachnoid hemorrhage simulations for preclinical learners are available.

Due to COVID-19 restrictions, access to clinical learning is limited. Virtual simulation of clinical encounters can be used to help fill this gap in clinical experience for preclinical students [9]. Neurology simulations are particularly well-suited to virtual platforms, as the diagnosis is heavily reliant on the correct interpretation of the history, which can be done virtually [10]. Portions of the neurological physical exam, such as speech, visual fields, pupil symmetry, facial weakness, and coordination, can also be performed over basic videoconferencing platforms [11].

Medical students are underrepresented in the meta-analysis of the efficacy of simulation as a learning tool [12]. While anecdotal evidence shows that medical learners enjoy simulation and feel that they are performing better, little work has been done that quantifies the impact of preclinical simulation education. Implementing a virtual neurology simulation in the preclinical years may allow for longitudinal studies to occur, which can examine the impact that preclinical simulation-based education has on patient outcomes. Additionally, the knowledge base surrounding the efficacy of virtual simulation shows low to modest positive effects of virtual simulation on clinical reasoning [13], results which can be augmented or contradicted using this scenario.

Solution

This simulation will provide the opportunity for preclinical students to (1) Show how to take a detailed headache history virtually, (2) Apply their clinical skills knowledge and complete a virtual neurological exam, (3) Produce a differential diagnosis for headache, including subarachnoid hemorrhage, (4) List investigations for patients with the presenting history, and (5) Recognize their own limitations and the need to call for help in a deteriorating patient scenario. By the end of the simulation, preclinical students should be able to recognize the red flag symptoms of a headache presenting to a virtual clinic and understand the need to refer the patient for emergent care.

This work has been presented in the Research Study DPP Program at the 21st International Meeting on Simulation in Healthcare on February 2, 2021, and will be available as a poster at the 2021 Canadian Conference on Medical Education from April 15 - July 20, 2021.

## Technical report

Learning objectives

By the end of the simulation and debrief, the student will be able to: (1) Demonstrate how to take a detailed headache history virtually; (2) Demonstrate how to do a virtual neurological exam; (3) List the differential diagnosis for headache, including subarachnoid hemorrhage; (4) List the appropriate investigations for patients with a subarachnoid hemorrhage; (5) Recognize their own limitations and the need to call for help in a deteriorating patient scenario.

Case

The patient is a 56-year-old, previously healthy woman who made an appointment at her family doctor’s office several hours after she worked out at the gym and experienced the “worst headache of her life.” The headache started after she fell on her treadmill at the gym that morning. She hit her head, but the headache was much worse than at other times she had fallen down. She reported that the pain included her whole head and neck, was of throbbing quality behind the eyes, and was gradually resolving from an intensity of 10/10 right after she fell 1.5 hours ago to a 7/10 at the time of presentation. Her pain was exacerbated by exercising and nothing relieved the pain. She had sensitivity to light and felt nauseous but had not vomited. She did not have a fever, sweats, or chills. There was no loss of consciousness.

The patient reported that she had fallen before while running, but she had never had a serious head trauma (i.e. concussion, loss of consciousness, etc.). She had had headaches before, but never anything serious, and she usually took acetaminophen for the pain. This was the most painful headache that she could recall. On history, she reported that her only medication was 12.5 mg of hydrochlorothiazide daily, and she had no known drug allergies. Her father died of myocardial infarction (MI) at 72, and her mother died of brain cancer at 76. One sister (49) had frequent migraines and her brother (57) was hypertensive. She reported drinking one triple shot of cappuccino every morning, and she had one this morning before her run.

Context 

The simulation is a virtual meeting between a patient and a preclinical clerk. The patient should be at home, on a video-based meeting platform such as Zoom, Skype, Webex, etc. Alternatively, the patient can call in from the phone, as this reflects the reality of how many patients interact with their doctors during times of restricted access, such as COVID-19 or rural settings. However, in the case of a phone call, the physical exam portion of the simulation should be omitted.

This context is appropriate because it is simultaneously removing distractions from the preclinical student, such as an unfamiliar simulation setting such as a hospital room or clinic room, which allows them to focus on the presenting symptoms of the patient, while additionally realistically demonstrating the challenges of providing remote healthcare. Since this is a skill that is likely to be needed in future clinical practice, running the simulation virtually addresses the practical issue of training students for a future of restricted clinical encounters while also enabling the simulation to be run in a low-resource manner.

In order to contribute to the growing knowledge base surrounding virtual simulation in medical education, this simulation will follow a similar structure to other virtual simulations in different disciplines [9]. The learner will log into a room with a patient, collect the history and any possible elements of the physical exam, and then the learner will be joined by a supervisor to present the case and inform the patient of next steps.

Inputs

This simulation requires a virtual video platform or conference calling, one standardized patient trained in the case and one evaluator/educator to observe the encounter, provide prompts if no action is taken by the student, and record the student’s approaches to the history taking and the virtual physical exam in order to provide feedback. Evaluator experience in virtual clinical encounters is beneficial but not necessary.

The preclinical student should have completed their preclinical neurology and head and neck instruction. The student will collect a focused neurological history and then complete the elements of the neurological exam that are possible to complete virtually such as asking about disturbance to taste/smell/hearing, asking the patient to demonstrate coordinated movements, asking about pain on neck flexion, etc. The aspects of the history and physical exam that the students are expected to perform can be found in Tables 1-2 and will be provided to the student beforehand. The evaluator will then ask the student to provide a differential diagnosis for the headache presentation and then ask the student to identify the best course of action.

**Table 1:** Information gathered during the focused history

Patient ID	Brenda Smithers, 56 y.o. female
Chief Complaint	Headache
History of Presenting Illness	The patient returned from the gym, where she fell on the treadmill and had sudden onset of a severe headache. She describes it as the “worst headache of her life” and she called the family doctor for a virtual appointment straight away. The pain is already subsiding. Patient reports photophobia. She is nauseated but hasn’t vomited. No fever, chills, or sweats. She didn’t take any medication. She was worried about taking something before coming to the clinic that might mask her symptoms. Character: Throbbing. Location: Frontal and bilateral. Onset: After exercise. Provoking factors: Exercise, fall on a treadmill. Palliating factors: None. Radiation: No. Associated signs and symptoms from a review of symptoms: Photophobia, nausea, stiff neck.
Past Medical and Surgical History	Hypertension, asthma as a child, hysterectomy in her 40s for menorrhagia
Medications	Hydrochlorothiazide 12.5 mg daily
Allergies	No known drug allergies (NKDA)
Family History	Father died of myocardial infarction (72), mother died of brain cancer (76), 1 brother, 1 sister, both well
Social History	Alcohol – one glass of wine per day. Non-smoker, no recreational drugs. Married, lives with husband. Elementary school teacher. No children. Hobbies: running, swimming, reading. Caffeine – 1 triple shot cappuccino every morning

**Table 2:** Virtual neurological physical exam findings The virtual testing methods were reproduced in part from Blue et al. [11].

Area of Interest	Virtual Testing Method	Findings on Exam
Taste and Smell	Ask about taste and smell disturbances	Nil
Speech	Evaluate fluency, comprehension, naming, repetition, reading, and writing	The patient becomes more confused as the exam progresses
Extraocular Movements	Ask the patient to look in the six cardinal positions of gaze. Ask the patient to fixate on the screen and move head left and right. Assess for nystagmus	The patient reports photophobia
Visual Acuity	Assess ability for the patient to read newsprint with each eye with the other eye covered	Normal
Facial muscles	Ask the patient to raise eyebrows, puff out cheeks, close eyes tightly, smile to assess for symmetry	Normal
Hearing	Ask the patient to rub fingers together by their ears and if they can hear it	Normal
Palate	Inspect for symmetric palate elevation	Normal
Shoulders	Assess for symmetric shoulder shrug	Normal
Sternocleidomastoid	Ask the patient to look all the way left, then all the way right	The patient reports neck stiffness
Tongue	Ask the patient to stick out their tongue, assess for deviation and fasciculations	Normal
Cerebellar Examination	Ask the patient to perform rapid alternating hand movements	Normal

Process

Pre-briefing

A pre-briefing information document will be provided to students prior to the simulation, which will provide a brief introduction to conducting a neurological examination (history and physical) virtually using the testing techniques and questions outlined in Tables 1-2. This information document will also include one to three references for further reading about the evaluation of headaches. We recommend Tabatabai and Swadron [14] as pre-reading for this simulation, however, each institution could tailor their document to reflect their curriculum.

A pre-briefing session will be held immediately before the simulation will clarify the learning objectives of the simulation, establish a “fiction contract” with participants, describe the logistics of the simulation, and reassure participants that there is a commitment to their learning and psychological safety, in line with the pre-simulation briefing guidelines set out by Rudolph et al. [15].

Scenario

After the pre-briefing session, the student will log onto a teleconferencing platform where a preceptor and a standardized patient will be waiting. The preceptor will ask the student to take a focused history and physical and report the details back, and then the preceptor will turn off their camera but stay in the meeting room to intervene if necessary. The student should obtain the information in Table 1 during their focused history (Objective 1).

**Figure 1:**
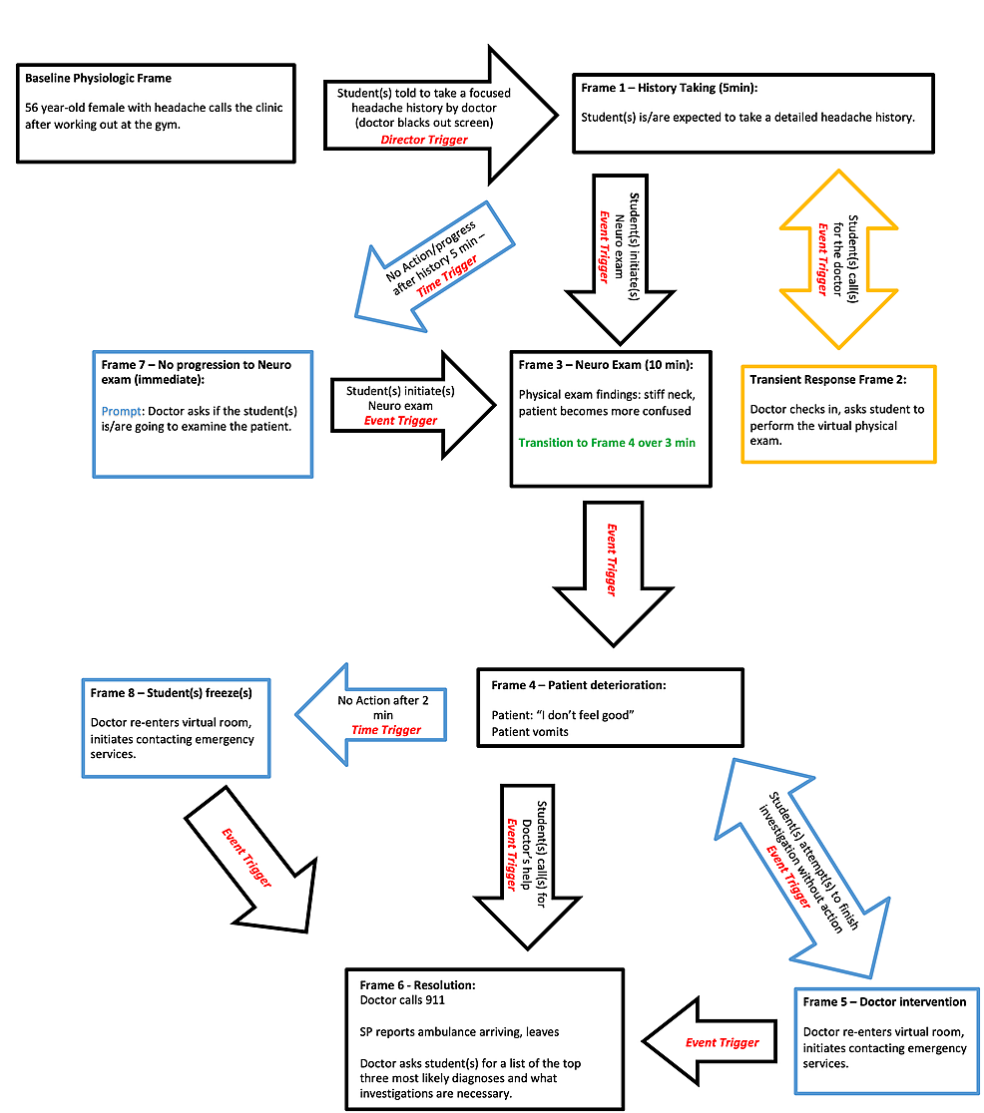
Virtual stimulation storyboard

The student then should move on to the virtual physical exam (Objective 2). The student should gather the information shown in Table 2. During the course of the physical exam, the patient will deteriorate on-screen and vomit near the end of the physical exam. After completing the virtual physical exam, the student should recognize the emergent nature of the situation due to the presence of red flag symptoms and ask for the preceptor to come back (Objective 5). The preceptor will turn on their video, then ask the standardized patient to call emergency services or offer to do it for them, and will ask the patient to remain on the video platform until help arrives. The standardized patient will report that emergency services have arrived, and the standardized patient will leave. The preceptor will then ask what the differential diagnosis is for the patient (Objective 3) and what the necessary investigations are (Objective 4). A detailed flowchart of the simulation, following the style suggested in O’Regan & Coombs-Thorne [16], is shown in Figure 1.

Feedback and Debriefing

Students will be debriefed virtually immediately following the simulation when the scenario is still fresh in their minds. 

There are currently no debriefing tools specifically designed for virtual simulation [17], therefore, the debriefer should use a method that they are experienced with and that has been validated for in an in-person simulation until an appropriate virtual simulation debriefing tool becomes available. We suggest the “Debriefing with Good Judgement” method created by Rudolph et al. [18], which focuses on delivering feedback using the Socratic method in order to create a psychologically safe environment to learn from one’s mistakes in the simulation [18]. It is meant to maximize the lessons the learner takes away without feeling making the learner feel unduly criticized. Since this simulation is targeted at early medical learners, and it is critically important to not only be cognizant of the learner’s skill level (ie. many preclinical learners will not have established approaches to making challenging diagnoses) but to refrain from creating an unfriendly learning environment that may discourage future participation in simulations.

The goal of the debriefing with good judgment is to challenge the cognitive frames through which the student evaluated the simulation to ensure that the student has good mental models for dealing with headaches in the future. The debriefer will approach the student’s mistakes (if any) with genuine puzzlement regarding the student’s thought and decision-making process and with respect for the learner and themselves. The debriefer will pair observations with inquiry in order to ask the student to explain why they took the actions they did at every step of the simulation and challenge the base assumptions that led the students to make their decisions.

The debriefing period will provide an opportunity for the evaluator to fill in clinical knowledge gaps for the preclinical learner so that they may be better prepared to address the challenges of diagnosing a subarachnoid hemorrhage in the future. It will also provide an opportunity for the experience debriefer to share their own frameworks for identifying challenging diagnoses.

Post-Scenario Didactics

A subarachnoid hemorrhage (SAH) occurs when blood moves into the subarachnoid space due to a compromise in a cerebral artery wall. It is commonly caused by a traumatic event or elevated blood pressure, which leads to the rupture of a preexisting brain aneurysm [1]. Many patients (20%-60%) present with what is known as a “thunderclap headache,” which is described as the worst of the patient’s life [1,19], though the quoted percentage of patients presenting with this symptom can be as high as 97% in the literature [3]. Additional symptoms include vomiting, photophobia, nuchal irritation, low-grade fever, and altered mental status [1].

As the blood diffuses into the subarachnoid space, symptoms may appear to resolve, which in combination with the broad differential diagnosis for SAH, which includes ischemic stroke, meningitis, primary headaches syndromes, venous thrombosis, and drug toxicity among others, can lead to SAH being missed entirely or misdiagnosed [1]. Subarachnoid hemorrhage is, therefore, considered one of the most difficult diagnoses for emergency medicine practitioners [3].

Students should be given an overview of the above information post-simulation in order to solidify their understanding of the presentation of the patient in the simulation. Students should also be advised of the appropriate diagnostic approach to take and the recommended intervention.

The gold standard of diagnosis is a non-contrast CT scan, which is 98% accurate within 12 hours of symptom onset [1]. The CT sensitivity drops to 76% after 48 hours after symptom onset to about 50% at one week [19]. If the CT is negative but SAH is strongly suspected due to symptoms, risk factors, such as age, sex, and lifestyle, family history, or patient history, a lumbar puncture is indicated, which should confirm the diagnosis of SAH if the condition is present [1]. It is also worth noting that the initial CT scan will be negative regardless of time since symptom onset for 2-5% of patients [19].

For the preclinical learners, it is most important to identify CT as the test that needs to be ordered for SAH since it is highly accurate but only if ordered within hours of the first presentation. It is also critical to recognize that a lumbar puncture would still need to be performed if the clinical suspicion of SAH is high and the CT scan is negative. The particulars of the treatment protocol for SAH are beyond the scope of this simulation, which is primarily targeted at developing recognition of red-flag headache symptoms and establishing the instinct to order further testing emergently.

Products/outcomes

The main outcome being evaluated is the learner’s perception of the effectiveness of their learning experience. After completing the simulation and the debrief, the students will be asked to complete the post-simulation survey given in the Appendix. The post-simulation survey includes questions that will qualitatively and quantitatively probe the students’ subjective experience of the simulation in order to fully understand the impact of the virtual simulation on the student’s learning experience.

The survey questions were adapted from the Simulation Effectiveness Tool - Modified (SET-M) [20], which has been found to be valid and reliable in nursing and medicine education. The SET-M is applicable to virtual simulations as well with slight modifications to the questions, which have been implemented in our survey [20]. The questions are scored on a three-point Likert scale and are designed to evaluate the learner’s perception of the effectiveness of the simulation. Results from the post-simulation survey can be analyzed to improve the scenario in subsequent trials. Further pre and post-simulation metrics can be added to this tool to determine the impact it may have on learner's knowledge or performance in neurology, but this is outside the scope of the tool provided in the appendix.

The last question in the survey provides an opportunity for the students to report any experiences that were not accurately or completely captured by the ranked questions. Thematic analysis can be undertaken using this question to identify areas of improvement for the simulation in the specific context of each school.

## Discussion

Developing a virtual subarachnoid hemorrhage simulation for preclinical learners fills a critical gap in the available simulation-based learning tools and enables preclinical learners to access clinical learning scenarios during times of limited access to hospitals. This simulation is accessible to institutions with limited access to high-fidelity simulation centers. While the post-simulation survey provided is intended to help institutions improve simulation delivery for their students, further evaluation tools could be used in addition to the one provided to longitudinally evaluate the impact of the simulation on pre-clinical student knowledge and skills, which would address a critical gap in the literature surrounding the efficacy of simulation-based education for preclinical learners.

This simulation is targeted at preclinical learners so it does not train students to intervene on their own in the case of subarachnoid hemorrhage and, by extension, other neurological emergencies. We feel that this is appropriate preparation for the clerkship environment; however, this tool is likely too limited for use in resident-level training. There are however many more available tools and simulations in neurology available for postgraduate medical learners, so we did not consider this to be a major gap.

This simulation could be adapted for use as an Objective Structured Clinical Examination (OSCE) using the metrics outlined in Tables 1 and 2 regarding the information that is expected to be gathered during the simulation by the student and the methods of obtaining that information in the physical exam. However, we did not undertake this adaptation, as evaluation of physical exam skills in a virtual environment is not currently common or validated practice in undergraduate medical education.

## Conclusions

Simulation-based learning can enhance medical students’ retention of material and confidence in clinical situations. A subarachnoid hemorrhage is considered a useful condition to teach using simulation due to the high mortality risk associated with a missed diagnosis. Virtual simulation resources are increasingly necessary due to restricted access to clinical learning experiences for preclinical students during the COVID-19 pandemic. In the future, one may expect that virtual clinical encounters may become more likely, further supplementing the need for the creation of virtual simulations for medical education. No virtual simulation-based educational tools exist to teach the diagnosis of subarachnoid hemorrhage to preclinical medical students, mirroring the overall lack of neurological simulation teaching tools. This could have an impact on future patient outcomes, as a subarachnoid hemorrhage is likely to appear during practice in the fields of family medicine, emergency medicine, or neurology, but is not likely to be observed during the core rotations.

Using virtual simulation-based learning to fill this gap in medical student knowledge may help mitigate future harm. Furthermore, the implementation of a virtual subarachnoid hemorrhage simulation and related feedback will build upon the existing literature surrounding the efficacy of virtual simulation as a training tool for preclinical medical students.

## Appendices

**Table 3:** Post-simulation survey Adapted from the Simulation Effectiveness Tool - Modified [20] for this simulation.

PREBRIEFING:	Strongly Agree (3)	Somewhat Agree (2)	Do Not Agree (1)
Prebriefing increased my confidence			
Prebriefing was beneficial to my learning			
SCENARIO:			
I am better prepared to respond to changes in my patient’s condition			
I developed a better understanding of the pathophysiology of subarachnoid hemorrhage			
I am more confident of my assessment skills			
I felt empowered to make clinical decisions			
I had the opportunity to practice my clinical decision-making skills			
I am more confident in my ability to prioritize care and investigations			
I am more confident in communicating with my patient			
I am more confident in my ability to report information to the health care team			
I am more confident in providing investigations that foster patient safety			
I am more confident in using evidence-based practice to provide care			
DEBRIEFING:			
Debriefing contributed to my learning			
Debriefing allowed me to communicate my feelings before focusing on the scenario			
Debriefing was valuable in helping me improve my clinical judgment			
Debriefing provided opportunities to self-reflect on my performance during simulation			
Debriefing was a constructive evaluation of the simulation			
What else would you like to say about today’s simulated clinical experience?			
